# Percutaneous treatment with the ‘valve-in-valve’ technique in congenitally corrected transposition of the great arteries and systemic atrioventricular valve with severe double lesion: case report

**DOI:** 10.1093/ehjcr/ytaf617

**Published:** 2025-11-28

**Authors:** Eliú David Pérez Nogales, Sandra Rodríguez Fuster, Elisabet Viera Reyes, Héctor Marrero Santiago, Francisco Jiménez Cabrera

**Affiliations:** Complejo Hospitalario Universitario Insular Materno Infantil, Las Palmas de Gran Canaria C.P. 35016, Spain; Complejo Hospitalario Universitario Insular Materno Infantil, Las Palmas de Gran Canaria C.P. 35016, Spain; Complejo Hospitalario Universitario Insular Materno Infantil, Las Palmas de Gran Canaria C.P. 35016, Spain; Complejo Hospitalario Universitario Insular Materno Infantil, Las Palmas de Gran Canaria C.P. 35016, Spain; Complejo Hospitalario Universitario Insular Materno Infantil, Las Palmas de Gran Canaria C.P. 35016, Spain

**Keywords:** Congenital heart disease (CHD), Interventional cardiology, Transposition of the great arteries (TGA), Adult congenital heart disease (ACHD), Percutaneous treatment, Case report

## Abstract

**Background:**

Congenitally corrected transposition of the great arteries (CCTGA) is a rare congenital heart disease characterized by atrioventricular (AV) and ventriculoarterial discordance, allowing near-normal circulation without early surgical correction. However, long-term complications such as valvular dysfunction, systemic right ventricular (RV) failure, and pulmonary hypertension are common. Management becomes particularly challenging in patients with prior interventions, where anatomical complexity and elevated surgical risk limit therapeutic options.

**Case summary:**

We present the case of a 47-year-old Chinese female diagnosed with CCTGA who had previously undergone surgical implantation of a bioprosthetic valve in the systemic AV position in her home country. She presented with significant deterioration in functional capacity, and imaging revealed severe dysfunction of the surgical prosthesis, characterized by severe mixed valvular disease, aneurysmal dilation of the left atrium, and severe systemic RV dysfunction. A multidisciplinary team assessment determined that the patient was at high surgical risk, leading to the decision to perform a percutaneous valve-in-valve implantation. An Edwards SAPIEN® 26 mm bioprosthetic valve was successfully implanted, yielding excellent haemodynamic results without complications. The patient was discharged 24 h post-procedure with significant clinical improvement.

**Discussion:**

Although percutaneous interventions for systemic tricuspid valve disease have been described, they typically involve native valves and remain anecdotal. To date, no published reports have documented valve-in-valve implantation in a systemic AV prosthesis in a patient with CCTGA. This case illustrates the feasibility and safety of such an approach in a high-risk patient, offering a valuable alternative to surgical replacement and potentially delaying the need for heart transplantation.

Learning points

- Percutaneous valve-in-valve implantation can be a feasible alternative to high-risk reoperation in patients with failing systemic atrioventricular prostheses in congenitally corrected transposition of the great arteries.
- In the absence of guideline recommendations, individualized strategies are essential for managing prosthetic valve degeneration in congenital heart disease.
- A multidisciplinary heart team approach enables safe, minimally invasive solutions that may delay heart transplantation in complex adult congenital anatomy.

## Summary figure

**Figure:**
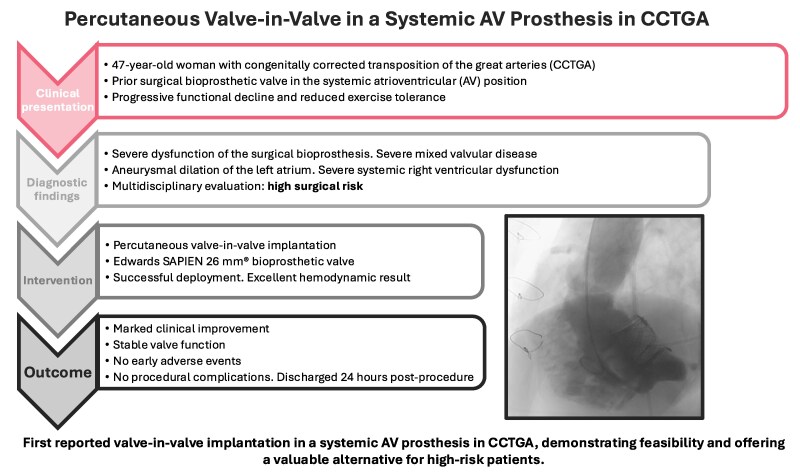


## Introduction

Congenitally corrected transposition of the great arteries (CCTGA) is a rare congenital heart disease (CHD) characterized by atrioventricular (AV) and ventriculoarterial discordance, allowing systemic and pulmonary circulation without the need for immediate surgical correction. However, in the long term, these patients are at high risk of developing complications, including valvular insufficiency, systemic right ventricular (RV) dysfunction, and pulmonary hypertension. Managing such cases can be challenging, particularly in patients who have undergone prior interventions, where treatment options may be limited by anatomical complexity and high surgical risk.

We report a CCTGA patient with prior systemic AV bioprosthesis, lost to follow-up abroad. Years later in Spain, she developed severe prosthetic dysfunction, mixed valvular disease, pulmonary hypertension, and systemic ventricular impairment. This complex case underscores the value of multidisciplinary care and the emerging role of percutaneous structural interventions to delay advanced therapies (such as heart transplantation) and reduce surgical risk in high-risk patients.

## Case report

We present the case of a 47-year-old woman born in China, referred to our hospital for evaluation of a previously treated CHD. She had undergone surgical intervention in 2005 in her home country; however, no prior medical records were available.

She worked as a waitress and was referred for dyspnoea on mild exertion (New York Heart Association (NYHA) III) without accompanying symptoms such as chest pain or palpitations.

**Figure 1:**
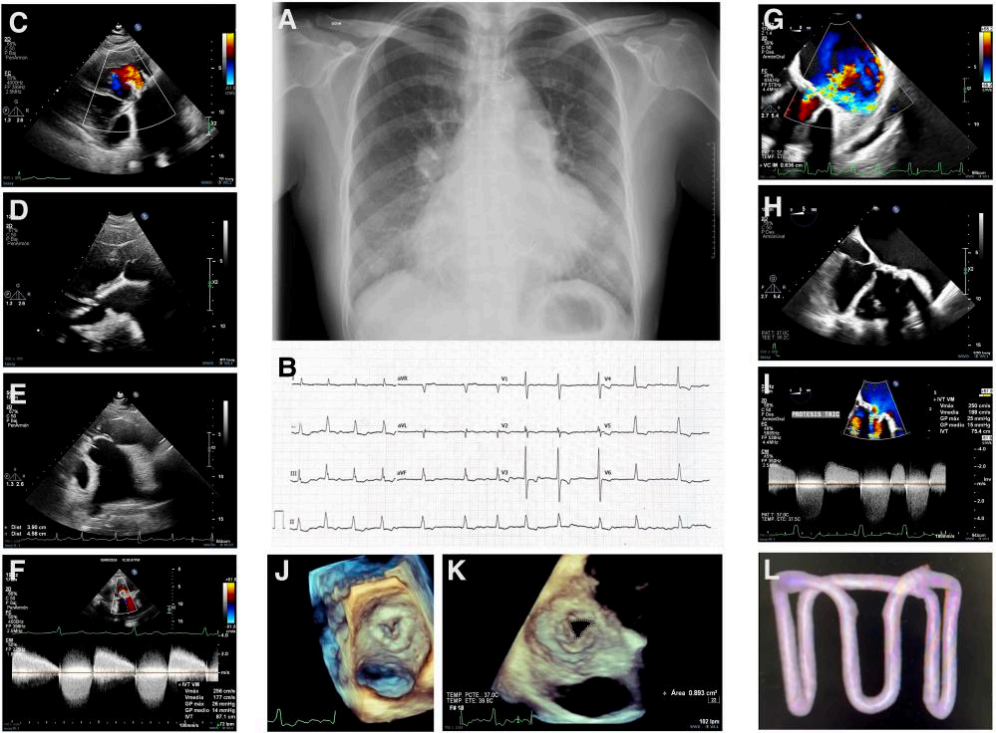
(*A*) Chest X-ray showing dextrocardia, pulmonary congestion, prosthetic valve structures in the systemic atrioventricular position, and surgical cerclage materials. (*B*) Electrocardiogram: atrial fibrillation with a controlled ventricular response. (*C–F*) Transthoracic echocardiographic views confirming atrioventricular and ventriculoarterial discordance consistent with congenitally corrected transposition of the great arteries. Severe mixed dysfunction of the systemic atrioventricular bioprosthesis is evident, with a mean gradient of 14–15 mmHg and severe regurgitation. Aneurysmal dilation of the left atrium (78 × 61 mm) and systemic right ventricular dysfunction are also noted. (*G–K*) Transoesophageal echocardiogram confirming severe stenosis of the systemic atrioventricular bioprosthesis (valve area 0.9 cm², Vmax 2.5 m/s, mean/peak gradient 15/25 mmHg) and severe intraprosthetic regurgitation. Subpulmonary atrioventricular valve shows minimal regurgitation, allowing estimation of a peak gradient of 45 mmHg. Pulmonary trunk is aneurysmally dilated with moderate pulmonary regurgitation. (*L*) Three-dimensional computed tomography reconstruction of the previously implanted bioprosthetic valve, suggesting an inverted aortic Hancock® valve, used to guide sizing and planning of percutaneous valve-in-valve implantation.

On physical examination, a grade V/VI systolic–diastolic murmur was auscultated at the mitral focus, with bilateral pulmonary basal crackles and a tendency towards hypotension. Chest X-ray (*Figure 1A*) revealed dextrocardia, signs of pulmonary congestion, prosthetic support structures in the systemic AV valve, and surgical cerclage materials.

An initial transthoracic echocardiogram was performed to clarify the underlying CHD and assess the cause of dyspnoea (*Figure 1C–F*). The findings confirmed dextrocardia with a right apex, AV and ventriculoarterial discordance, and CCTGA. The bioprosthetic valve in the systemic AV position showed severe obstruction [mean gradient (MG) of 14–15 mmHg] and severe regurgitation, associated with aneurysmal dilation of the left atrium (78 × 61 mm). The patient also presented severe systemic ventricular dysfunction, marked dilation of the pulmonary trunk (maximum diameter of 54 mm), and atrial fibrillation (*Figure 1B*).

Given the severe prosthetic dysfunction, the patient was hospitalized for decongestive therapy, further evaluation, and therapeutic planning. Laboratory results showed N-terminal pro-B-type natriuretic peptide (NT-ProBNP) of 2500 pg/ml and normal renal, hepatic, and thyroid function.

The transoesophageal echocardiogram (TEE) confirmed previous findings (*Figure 1G–K*): severe valvular stenosis with a three-dimensional valve area of 0.9 cm² (Vmax 2.5 m/s, mean/peak gradient 15/25 mmHg) and severe intraprosthetic regurgitation. There was minimal regurgitation of the subpulmonary AV valve, allowing the estimation of a peak gradient of 45 mmHg. The pulmonary trunk showed aneurysmal dilation with moderate pulmonary regurgitation. Cardiac magnetic resonance imaging showed a dilated systemic ventricle with severe trabeculation and moderate dysfunction (ejection fraction (EF) 35%, end-diastolic volume (EDV) 132 ml/m², and end-systolic volume (ESV) 72 ml/m²). The subpulmonary ventricle had better contractility (EF 43%, EDV 103 ml/m², and ESV 58 ml/m²). There were no areas of late gadolinium enhancement.

After achieving euvolemia, right and left heart catheterization was performed, confirming normal coronary arteries, elevated pulmonary capillary wedge pressure (PCWP) of 30 mmHg, mean pulmonary artery pressure of 34 mmHg, pulmonary vascular resistance (PVR) of 1.2 Wood units, and transpulmonary gradient of 4 mmHg, all findings consistent with moderate post-capillary pulmonary hypertension. The cardiac output was reduced to 2 l/min/m². The MG of the systemic AV valve was 25 mmHg, with a valve area of 0.5 cm².

A multidisciplinary conference was convened, considering the patient to be at extremely high surgical risk, mainly due to moderate-to-severe systemic ventricular dysfunction. Initially, evaluation for heart transplantation was proposed, with plans to reassess her candidacy in a future committee meeting. However, given her age and the long-term implications of heart transplantation, an alternative strategy was considered to prolong her native cardiac function through percutaneous valve implantation using a valve-in-valve approach within the systemic AV prosthesis.

A three-dimensional computed tomography reconstruction of the bioprosthetic valve (*Figure 1L*) implanted in 2005 suggested an inverted aortic bioprosthesis, possibly Hancock® (height of 21.4 mm, external diameter of 37.6 × 34.4 mm, and internal of 21.2 × 20.3 mm). Ultimately, an Edwards SAPIEN® 26 mm valve was implanted under fluoroscopic and TEE guidance via left femoral arterial and right femoral venous access, with transseptal puncture guided by TEE. A Nitrix guidewire was advanced from the right atrium to the left atrium, then into the systemic ventricle and the aorta. Using a JR4 catheter, the guidewire was exchanged for a 0.035 mm Teflon-coated guide, followed by the deployment of a 25 mm loop snare to establish a veno-arterial circuit. The interatrial septum was dilated with a 10 mm balloon, and the native mitral valve was pre-dilated with a 14 mm balloon before successfully implanting the Edwards SAPIEN® 26 mm valve (Supplementary  *Videos S1–S3*).

The final outcome was excellent, with no periprosthetic regurgitation and a peak gradient of 4 mmHg. Arterial access was closed with AngioSeal®, while venous access was secured with a suture. The patient was admitted to the intensive care unit without complications and was discharged to the general ward within 24 h. A transthoracic echocardiogram performed 36 h post-implantation confirmed correct prosthetic positioning, good leaflet mobility, and a transprosthetic MG of 5 mmHg without regurgitation, with a small atrial septal defect secondary to the transseptal puncture.

Upon initial evaluation, the patient was receiving anticoagulation with acenocoumarol along with ramipril 2.5 mg daily. Due to symptomatic heart failure and signs of congestion, intravenous furosemide was administered until euvolemia was achieved. Hypotension limited the initiation of prognostic heart failure therapies; however, low-dose dapagliflozin (10 mg once daily), spironolactone (25 mg every other day), and bisoprolol (2.5 mg twice daily) were successfully introduced, the latter also aiding in rate control for atrial fibrillation. Following valve-in-valve implantation, the patient experienced modest improvement in blood pressure, allowing for outpatient titration of sacubitril/valsartan (24/26 mg twice daily).

The patient showed rapid symptomatic improvement, enabling optimization of heart failure management despite limited evidence in systemic RV dysfunction. NT-ProBNP dropped to 600 pg/ml at discharge. At 1-month follow-up, she reported marked functional improvement, had resumed work as a waitress, and performed well on treadmill testing, completing 6 min of exercise and reaching seven metabolic equivalents (METs).

## Discussion

CCTGA is a rare and complex condition. Although many patients remain asymptomatic into adulthood, complications such as systemic ventricular dysfunction and AV valve regurgitation often emerge later, posing therapeutic challenges. Current European Society of Cardiology (ESC) guidelines recommend surgical valve replacement (as repair is usually unfeasible) in cases of severe symptomatic tricuspid regurgitation or asymptomatic patients with progressive systemic RV dilation or dysfunction (Class IIaC or IIbC if systemic RV function is below 40%).^1^

Our patient had previously undergone surgical AV valve replacement, with a bioprosthetic valve now exhibiting severe mixed dysfunction. No specific guideline recommendations for managing such cases exist.

Although percutaneous intervention for systemic tricuspid valve disease has been described, most reports focus on native valve treatment, which remains anecdotal^2,3^ or small series^4^ supporting procedural feasibility and safety. However, no previous reports have documented percutaneous treatment of a deteriorated systemic AV prosthesis.

This case was particularly challenging due to her high surgical risk, associated with factors such as NYHA II-III functional class, atrial fibrillation, and a systemic RV EF of 35%, although pulmonary pressures were not prohibitive (mean pulmonary artery pressure (PAP) 34 mmHg). Other therapies, such as systemic ventricular resynchronization,^5^ were initially ruled out due to severe valvular dysfunction, with consideration for future intervention if ventricular function failed to improve post-implantation.

The strategy of percutaneous valve-in-valve implantation was chosen to minimize procedural risk while proving to be a *safe and feasible* approach that successfully delayed the need for heart transplantation, offering significant benefits in terms of prolonged survival and improved quality of life. However, this approach carries specific risks, including prosthesis malposition, paravalvular leak, valve thrombosis, and potential for systemic ventricular outflow tract obstruction, especially in anatomically abnormal hearts. Valve-in-valve procedures, while less invasive than redo surgery, may limit future options due to reduced effective orifice area, elevated transvalvular gradients, and the potential for patient-prosthesis mismatch, especially if the initial bioprosthesis was small. The durability of transcatheter valves in the systemic position is not well established, and repeated percutaneous interventions may be technically unfeasible. Additionally, the cumulative risk of arrhythmias, conduction disturbances, and endocarditis increases with each intervention. This case highlights the importance of a multidisciplinary approach and underscores the emerging value of percutaneous techniques as a viable option for managing CHD in high-risk patients.

## Lead author biography


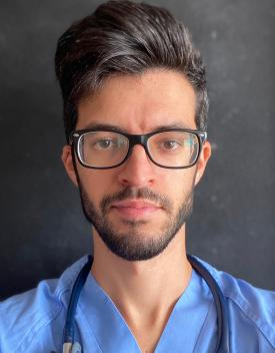


Dr Eliú Nogales obtained his medical degree from the University of Las Palmas de Gran Canaria. He completed his residency in Cardiology at Hospital Insular de Gran Canaria, followed by a Master’s degree in Cardiac Imaging from the University of Murcia. He pursued further training in adult congenital heart disease at Hospital Universitario La Paz (Madrid) and is currently undertaking doctoral studies in this field. His main areas of interest include congenital cardiology, advanced cardiac imaging, and structural interventions in complex anatomy.

## Supplementary Material

ytaf617_Supplementary_Data

## Data Availability

All data supporting the findings of this case report are included within the article. Additional clinical materials—including transthoracic and transoesophageal echocardiographic studies, imaging videos, and procedural documentation—are available from the corresponding author upon reasonable request.
